# A Systematic Framework for Analyzing Patient-Generated Narrative Data: Protocol for a Content Analysis

**DOI:** 10.2196/13914

**Published:** 2019-08-26

**Authors:** Maryam Zolnoori, Joyce E Balls-Berry, Tabetha A Brockman, Christi A Patten, Ming Huang, Lixia Yao

**Affiliations:** 1 Department of Health Sciences Research Mayo Clinic Rochester, MN United States; 2 College of Medicine and Science Mayo Clinic Rochester, MN United States; 3 Community Engagement Program Center for Clinical and Translational Science Mayo Clinic Rochester, MN United States; 4 Department of Psychiatry and Psychology Mayo Clinic Rochester, MN United States

**Keywords:** social media, online social networking, patient-centered care, qualitative research, framework method, inductive approach, deductive approach, text mining

## Abstract

**Background:**

Patient narrative data in online health care forums (communities) are receiving increasing attention from the scientific community for implementing patient-centered care. Natural language processing (NLP) methods are gaining more and more attention because of the enormous data volume. However, state-of-the-art NLP still cannot meet the need of high-resolution analysis of patients’ narratives. Manual qualitative analysis still plays a pivotal role in answering complicated research questions from analyzing patient narratives.

**Objective:**

This study aimed to develop a systematic framework for qualitative analysis of patient-generated narratives in online health care forums.

**Methods:**

Our systematic framework consists of 4 phases: (1) data collection, (2) data preparation, (3) content analysis, and (4) interpretation of the results. Data collection and data preparation phases are constructed based on text mining methods for identifying appropriate online health forums for data collection, differentiating posts of patients from other stakeholders, protecting patients’ privacy, sampling, and choosing the unit of analysis. Content analysis phase is built on the framework method, which facilitates and accelerates the identification of patterns and themes by an interdisciplinary research team. In the end, the focus of interpretation of the results phase is to measure the data quality and interpret the findings regarding the dimensions and aspects of patients’ experiences and concerns in their original contexts.

**Results:**

We demonstrated the usability of the proposed systematic framework using 2 case studies: one on determining factors affecting patients’ attitudes toward antidepressants and another on identifying the disease management strategies in patient with diabetes facing financial difficulties. The framework provides a clear step-by-step process for systematic content analysis of patient narratives and produces high-quality structured results that can be used for describing patterns or regularities in patients’ experiences, generating and testing hypotheses, and identifying areas of improvement in the health care systems.

**Conclusions:**

The systematic framework is a rigorous and standardized method for qualitative analysis of patient narratives. Findings obtained through such a process indicate authentic dimensions and aspects of patient experiences and shed light on patients’ concerns, needs, preferences, and values, which are the core of patient-centered care.

**International Registered Report Identifier (IRRID):**

RR1-10.2196/13914

## Introduction

Online health forums (communities) are increasingly accessible and convenient platforms for patients and caregivers to share health care experiences together with concerns of diagnosis, treatment, and outcomes. Almost 30% of the US population actively share and discuss health-related experiences on various online health forums, such as askapatient.com, patientslikeme.org, and dailystrength.org [1]. Patient-reported health care experiences in a narrative format on the internet are a valuable data source for implementing patient-centered care [2].

There are 2 methods for analyzing patient-generated narrative data: natural language processing (NLP) and qualitative content analysis. NLP is a set of methods and techniques to process human language components, such as identifying sentence structure and recognizing sentence meaning as humans do [3]. NLP algorithms build on statistical methods to infer patterns in data. The algorithms usually need an annotated dataset (train dataset) for learning meaningful patterns and concepts to make predictions about new data [4]. In the context of patients’ narratives, NLP is mostly used for clustering health-related messages [5], identifying the topics of concern [6], and recognizing the opinions and sentiments of patients toward different topics. With the fast-growing volume of patient-generated narratives in online health forums, NLP has been gaining increasing attention. However, the available NLP techniques are not able to provide a high-quality understanding of the text, such as identifying patients’ functional problems because of colloquial language, word ambiguity, and layperson terms [7,8].

The second method for analysis of patient-generated narratives is qualitative content analysis. Qualitative content analysis is a research method designed to identify the thematic structure of text documents by subjectively interpreting the context of the text [9]. This process is conducted through a systematic classification process of coding and identifying themes and patterns in the text. Unlike the NLP algorithms, this method can provide a deep insight into textual data for identifying patterns and various aspects of the textual content. However, this method is usually applied on a small sample of data because of the manual process of analysis. In the context of patient narrative data, qualitative content analysis has been used to identify meaningful patterns and themes in patients’ discussions in online health care forums to answer complex research questions such as factors affecting patients’ attitudes toward antidepressants and contributions of MD Anderson’s Facebook group to patient’s cancer experience.

In this study, we propose an efficient and cost-effective systematic framework built on text mining and qualitative content analysis approaches for analyzing patients’ narratives. This framework comprises 4 phases: (1) data collection, (2) data preparation, (3) content analysis, and (4) interpretation of findings. Data collection and data preparation phases utilize text mining methods to facilitate and accelerate the process of data collection and preparation for content analysis. The content analysis phase utilizes the framework method [10] and the deductive-inductive approach to facilitate the process of data analysis and interpretation of the findings. Overall, the proposed systematic framework offers a comprehensive approach to identify dimensions and aspects of patients’ narratives in online health care forums in an efficient manner.

In this study, we first provide a brief introduction to the framework method [10] and content analysis and theme generation approaches with examples from research conducted using patient-generated narratives. Next, we describe the proposed framework and demonstrate its applicability for analysis of patient narratives using 2 case studies: one on determining factors affecting patients’ attitudes toward antidepressants using data from askapatient.com [11] and another on identifying the disease management strategies in patients with diabetes facing financial difficulties using data from 4 diabetes-focused forums.

### The Framework Method for Content Analysis

**Figure 1:**
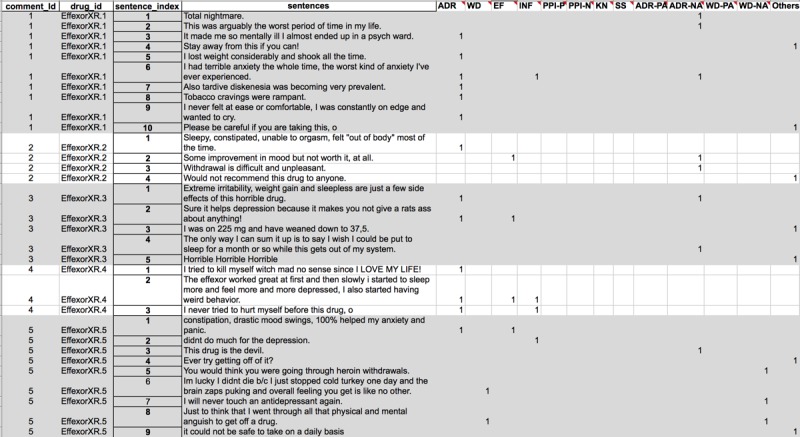
Structure of the framework method. Sentences from patient posts (unit of analysis) organized in the rows and themes (adverse drug reactions) organized in the columns. Value “1” indicates to which theme a sentence was assigned.

The framework method was developed in the qualitative research unit at the National Centre for Social Research in the United Kingdom in 1980 for analyzing narrative data related to policy [10] and later adopted in other fields, including health care. It identifies themes in data systematically, where each theme represents a semantic topic. More specifically, the framework method builds a matrix structure with each piece of narrative data (eg, a patient post or a sentence) stored in a row and each identified theme in a column. Using this structure, researchers can cluster narrative data around identified themes and identify the relationships between themes (see Figure 1). This framework provides a holistic, descriptive picture of a reasonably large sample of data.

Themes in the framework method are generated through deductive, inductive, or a combination of the deductive-inductive approach. The selected approach depends on the research question, the study’s aim, and the available knowledge about the phenomena under study. The deductive approach is used when the aim of the study is to retest the existing model, concept, or knowledge in a new context [12]. In the context of patient narratives, Latvala et al and Kasila et al used the inductive approach to identify meaningful concepts [13,14]. When the available knowledge about phenomena under study is limited, the inductive approach is used to identify new themes. For example, Zolnoori et al, Hilliard, and Sutton et al used the inductive approach to identify meaningful patterns and themes in patients’ discussions in online health care forums [15-17]. Finally, the deductive-inductive approach is used when researchers wish to test the existing knowledge in a new context and leave space to discover new aspects of the data that were not covered by existing knowledge. For example, Zolnoori et al used the deductive-inductive approach to test hypotheses concerning patients’ attitudes toward antidepressants using patients’ drug reviews in askapatient.com [11].

## Methods

### Overview

**Figure 2:**
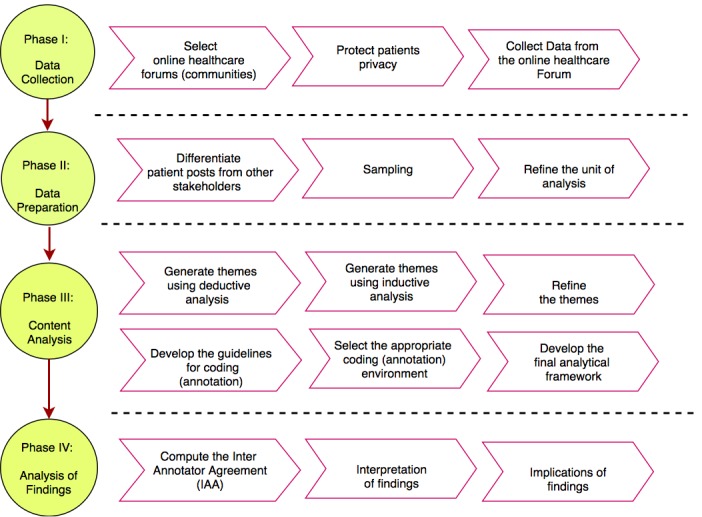
A schematic view of the systematic framework designed for content analysis of patient narrative data.

Building on our previous experiences and lessons learned from the content analysis of patients’ narratives, we propose a systematic framework to address the subjective nature of patients’ narratives [11,18-20]. This framework integrates text mining methods, the framework method, and deductive-inductive approach for content analysis and is composed of 4 main phases: (1) data collection, (2) data preparation, (3) content analysis, and (4) interpretation of the results. During the data collection phase, relevant health forums are selected regarding the aim of the study. The data preparation phase uses text mining methods for (1) distinguishing patients’ comments from other stakeholders (caregivers and clinicians), (2) sampling patients’ narratives, and (3) preparing the unit of analysis. The focus of content analysis phase is on the framework method and the deductive-inductive approach to generate a structured matrix that presents various aspects of patients’ narratives. Finally, the focus of interpretation phase is on measuring quality criteria, validation, and interpretation of findings. Figure 2 shows a schematic view of the systematic framework.

In the following sections, we demonstrate the implementation of each step of this systematic framework using 2 studies: (1) a case study that identified factors affecting patients attitudes toward antidepressants; we use the short title *attitude to antidepressants* to refer to this case study [11] and (2) a case study that analyzed the strategies and solutions of patients with diabetes facing financial difficulties to access medications and supplies; we use the short title *access to diabetes medications* to refer to this case study.

### Phase I: Data Collection

The data collection phase of the systematic framework included selecting health forums, protecting patients’ privacy, and collecting data from these health forums.

#### Selection Health Forums for the Study

Health forums vary in covering patients’ experiences with the health care system. Researchers can select a health care forum for their study using (1) description of the forum or community, (2) initial analysis of randomly selected posts, and (3) analysis of medical concepts using text mining tools such as MetaMap [21] or clinical Text Analysis and Knowledge Extraction System [22]. Researchers may choose multiple forums for a study to reduce the risk of potential bias in the findings that may occur because of an unbalanced sampling of patient experiences in a health forum. However, determining the number of forums depends on the aim of the study and the level of heterogeneity in patient experiences. For example, for the study *attitude to antidepressants*, we used only 1 forum askapatient.com because our initial content analysis using MetaMap [17] showed that this forum covers a wide range of antidepressants’ side effects and potential factors affecting patients’ attitudes toward antidepressants.

#### Patients’ Privacy in the Health Forums

Although data in the forums are mostly anonymous and publicly available, further protection of patient’s privacy and requesting permission from owners of the data collection are recommended. Researchers need to submit the institutional review board’s (IRB’s) study approval to the affiliated institute. The IRB submission usually receives an exemption. In addition, to further protect patient privacy, deidentification of the data is recommended. For example, in both projects, we formulated regular expressions to eliminate emails, phone numbers, and URLs from posts. For the project *attitude to antidepressants*, we sought IRB approval through the University of Wisconsin-Milwaukee, and for the project *access to diabetes medications*, we sought IRB approval through the Mayo Clinic.

#### Data Collection From an Online Health Forum

Patient posts in the online health forums are mostly stored in the HTML format. To collect these data, the research team may use the application programming interface (API) specifically developed for the forum or community. If the API is not available, the research team may customize the existing open-source Web crawlers or develop a new one to collect data. For example, we used Beautiful Soup [23], a python package for parsing HTML and XML documents, to develop a Web crawler. Please see Multimedia Appendix 1 for the definition of HTML, API, Web crawler, Python package, and XML.

### Phase II: Data Preparation

Data preparation phase consists of 3 steps: differentiating patient posts from other stakeholders, sampling, and defining the unit of analysis.

#### Differentiating Patient Posts From Other Stakeholders

Patients’ interests and perspectives on treatment are different from that of clinicians and caregivers who share their experiences and concerns for their patients in online health forums. Distinguishing patients’ experiences from other stakeholders can be achieved by utilizing text mining approaches such as unsupervised algorithms for text clustering [24] (eg, density-based spatial clustering of applications with noise, expectation-maximization, or agglomerative hierarchical clustering). For example, Lu et al leveraged the expectation-maximization algorithm for differentiation between patient posts from caregivers and clinicians [5]. To improve the performance of the clustering algorithms, selecting a proper feature such as writing style–based features (eg, lexical and syntactic) [25,26] and content-specific features (eg, kinship terminology, Unified Medical Language System [UMLS] semantic types and concept IDs, and n-grams) [26,27] is useful. See Multimedia Appendix 2 for more information about kinship terminology and UMLS.

#### Sampling

Having a representative sample of online forums content is pivotal for statistical reliability and generalizability of the findings. To increase the likelihood of having a representative sample, the research team may utilize retrieval methods such as phrase-based vector space model [28,29] or knowledge-based query expansion method [30]. The retrieval methods are particularly useful when the forums do not have a built-in system for filtering specific information, and the content covers a wide range of patients’ experience.

If the size of retrieved relevant patient posts is extremely large, probability sampling methods (such as simple random sampling, stratified random sampling, or cluster sampling) are useful to obtain a robust sample size *[31].* If the document retrieval methods do not retrieve relevant patient posts for the research question, the research team may use nonprobabilistic samplings, such as *convenience sampling or judgmental sampling [31].*

Determining the sample size is another concern in content analysis studies. There is no single formula for determining the sample size. The size of the sample is a factor of time and financial sources and data heterogeneity. Researchers may use the standard sampling formula for computing the sample size [32]. As an example of sample size calculation, please see Multimedia Appendix 3 [33,34].

#### Defining the Unit of Analysis

A unit of analysis is the smallest unit in the data sample containing information regarding the research question. Graneheim et al discussed that the unit of analysis should be large enough to convey a whole perspective and small enough to be kept in mind as a context for meaning unit during the analysis process [35].

For both case studies, the initial analysis showed that patients’ comments were composed of multiple sentences that covered various dimensions and aspects of experiences and concerns. Therefore, we used sentences as the unit of analysis. In addition, data analysis at the level of sentences ensured that no important segment of patient narratives was missed. Splitting patient posts into sentences is not an easy task because of colloquial language and grammatical and punctuation errors. Therefore, we preprocessed the data to remove noisy patterns and then split the patient posts into sentences using open-source Natural Language Toolkit [36]. Multimedia Appendix 4 shows examples of regular expression codes we used to handle the grammatical errors in patient posts.

### Phase III: Content Analysis

After preparing the patient posts, the next step is on defining themes for content analysis. The framework method allows different approaches for generating themes: deductive, inductive, and combination of deductive-inductive. In this section, we illustrate the step-by-step procedure of generating themes using deductive-inductive approach for the 2 case studies. This approach allowed us to retest the available knowledge in the literature in the context of patient narratives while leaving space for discovering new aspects of the patient experiences in online health forums.

In this section, we explain the process of generating themes for the case study *attitude to antidepressants*. The process of generating themes for the case study *access to diabetes medications* is presented in Multimedia Appendix 5

#### Generating Themes Using Deductive Analysis

**Figure 3:**
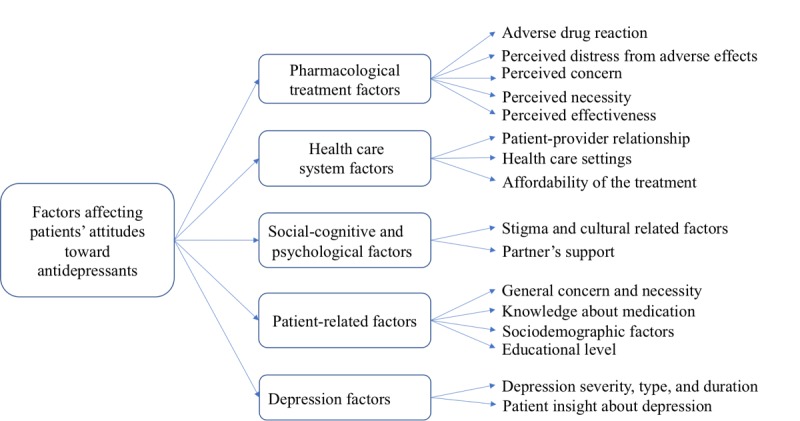
Generated themes using deductive approach for the case study attitudes to antidepressants.

Our literature review showed that existing knowledge in the literature is useful for generating themes to analyze and summarize patient experiences with antidepressants in online forums. Accordingly, we conducted a systematic literature review to identify significant factors affecting patients’ attitudes toward antidepressants. We identified 5 main themes including pharmacological treatment, health care system, social-cognitive and psychological factors, patient-related factors, and depression that influence patients’ attitudes toward antidepressants. For each theme, we identified subthemes. Figure 3 shows the themes and subthemes generated using deductive analysis.

To start coding patient posts using the predefined themes, developing guidelines with clear operational definitions for each theme is necessary. Operational definitions should include well-defined statements with explicit inclusion and exclusion criteria describing the segment of a text assigned to a theme. Each statement must accompany 1 or more examples extracted from patient posts. Table 1 provides an example of operational definitions of themes for the case study *attitude to antidepressants*. Multimedia Appendix 6 includes the operational definitions for all predefined themes of the study *attitude to antidepressants*.

Themes generated in deductive approach were used for generating the initial analytical framework. We constructed the framework by organizing predefined themes in the columns and sentences of patient posts (unit of analysis) in the rows. Each patient post was split into sentences and identified using post ID and sentence index indicating the position of the sentence in the patient post. Figure 1 shows the structure of the analytical framework for the case study *attitude to antidepressants.*

**Table 1:** Example of operational definition for the themes for the project *patient attitudes toward antidepressants* and category.

Pharmacological treatment factors (predefined codes)	Description
Perceived effectiveness	The patient’s subjective assessment of antidepressant helpfulness in the reduction of depression symptoms, enhancing emotional and cognitive functionalities, and overall, enhancing life quality. Example: *Anyway, my life is on track, I have nothing to be depressed or sad about.*
Side effects	Any adverse reactions that the patient reports as adverse reactions to antidepressants intake. Antidepressants’ adverse reactions may include physiological side effects, emotional syndromes, cognitive impairment, and limitations on daily functioning and quality of life. Example: *Typical with Effexor XR- Dizzy, jaw tight, teeth grinding.*

#### Generating Themes Using Inductive Analysis

As patients in the forums have the freedom to anonymously share their experiences and concerns in the lay language without any limitations, it is likely that patient posts include information that may not fit into the predefined themes in the initial analytical framework. Therefore, in this step, although we coded sentences (of patient posts) using predefined themes, we used the inductive approach to generate new themes for sentences that could not be assigned to the predefined themes.

It is not necessary to use the whole sample for inductive analysis. Researchers may select a random portion of the sample (eg, 30%), regarding the availability of resources, size of a sample, and the level of heterogeneity in patient narratives. For example, in the study *attitude to antidepressants*, we created a subsample by randomly selecting 33.33% (300/900) of the posts for inductive analysis. To identify new themes, 4 coders read the sentences in the subsample and assigned them to proper themes by following the guidelines. The meaningful sentence that could not fit into predefined themes was discussed in our regular team meetings for new themes. For example, we could not assign this sentence *I weaned slowly and I feel nauseous a lot* to any predefined themes to any available themes, so we generated a new theme named *withdrawal symptom*. We created a theme named *Not-applicable* that contained sentences without any meaningful information related to the research question. For example, *Feel free to email me about Effexor* does not reflect any information about the patient’s attitude toward antidepressants.

**Figure 4:**
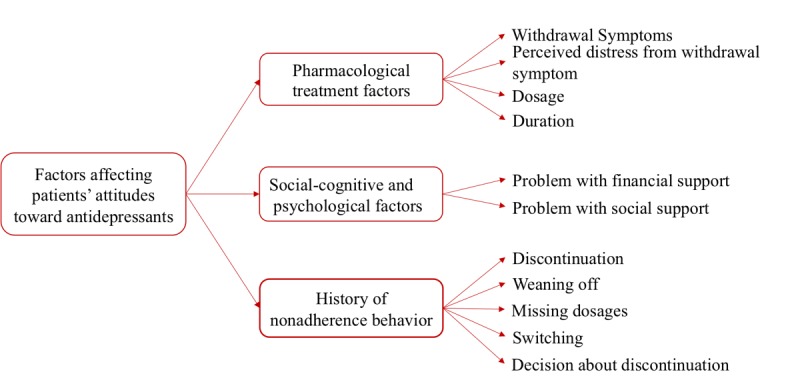
Generated themes using inductive approach for the case study “attitude to antidepressants”.

Figure 4 shows new themes generated using the inductive approach for the project attitude to antidepressants. Multimedia Appendix 7 includes all the generated new themes with the examples from patient posts for this study.

#### Refining the Themes

Some of the themes generated in deductive approach may not fit into patient posts. For example, we could not find any sentences in the subsample of the study *attitude to antidepressants* to assign to the theme *educational level*. In addition, some of the new themes developed through inductive approach may fit into a few patient posts. For example, nearly 1.9% (17/892) of the patient posts in the subsample had information related to *problem with financial support;* therefore, we removed the theme from the list.

Themes generated using inductive and deductive approaches need to be refined before developing the final analytical framework. Theme refining can be conducted by creating rules such as setting a threshold on the number of sentences that should be assigned to a theme. For example, for the study *attitude toward antidepressants*, we set the threshold on 5%, that is, if a theme included less than 5% of sentences in the subsample, we excluded them from the list of themes. The value of the threshold depends on the importance of the rare or uncommon themes (patterns) in the dataset for the research team. For example, for the case study *access to diabetes medications*, we did not consider any threshold because of the importance of the rare information that patients reported to get access to medications. But for the case study *attitude toward antidepressants*, it was not possible to make any conclusion based on the rare themes in the dataset, that is, to measure the association between rare themes and patients’ attitudes. Therefore, our research team set the threshold on 5% after the initial analysis of 30% of the dataset.

Multimedia Appendix 8 includes all the rules generated to refine themes for the study *attitude to antidepressants.*

#### Developing the Guideline for Coding

To maintain consistency and uniformity of coding patient posts using the final themes across the sample, developing guidelines are necessary. Guidelines should include the aim of the project, operational definition of the themes with specific examples from patient posts, and inclusion and exclusion criteria for assigning a unit of analysis to themes. Operational definition for a theme should include a clear and precise statement that enables the annotators to recognize a segment of patient post that fit the theme. For example, theme *adverse drug reaction* (ADR) in the study *attitude to antidepressants* defined as *any signs or symptoms that patients experienced after drug consumption and explicitly and certainly were linked to the drug consumption* includes criteria to decide what is ADR by emphasizing on the time of occurrence of the sign or system, that is, *after drug consumption*. The definition also used the terms *explicitly* and *certainly* to exclude any vague or uncertain statements from the patient post. Vague or ambiguous operational definition increases the need for text interpretation, raises the risk of observational error in coding, and consequently results in low interannotator agreement (IAA).

The guidelines should also include instruction on coding the unit of analysis using themes. For example, whether the unit of analysis can be assigned to more than 1 theme or whether the unit of analysis should be interpreted in the context of the patient posts are important questions. Clear answers to these questions can certainly facilitate the process of coding and increase the quality of generated structured data. Finally, it would be useful if coding guidelines include the list of qualifications for hiring annotators and the estimated time for training. Multimedia Appendix 9 contains the guidelines that we developed for the study *access to diabetes medications.*

#### Selecting Appropriate Coding Environment

The research team should select a coding environment that facilitates construction of the analytical framework and the process of coding. For both case studies explained in this study, we used a spreadsheet to construct the analytical framework (see Figure 1). Annotators (coders) could assign a sentence to a theme by inserting 1 in the intersection of the sentence and the theme. However, if the unit of analysis is a phrase or a word, the spreadsheet may not be a convenient tool for constructing the analytical framework and annotating process. In this case, tools such as *Brat* [37] or *MAE* [38] are helpful. But if the unit of analysis can be defined as any segment of text (patient post), tools that were specifically designed for qualitative data analysis, such as NVivo (a qualitative data analysis (QDA) computer software package produced by QSR International) [39], would be more useful.

#### Developing the Final Analytical Framework

Overall, the final themes should meet the following criteria: (1) valid—themes should accurately reflect what is being measured, (2) mutually exclusive—themes should not overlap between operational definitions, and (3) exhaustive—themes should cover all the aspects of the data related to the research question. *All* aspect of the data means identifying all the dimensions of the dataset that provide meaningful information for the research question.

**Figure 5:**
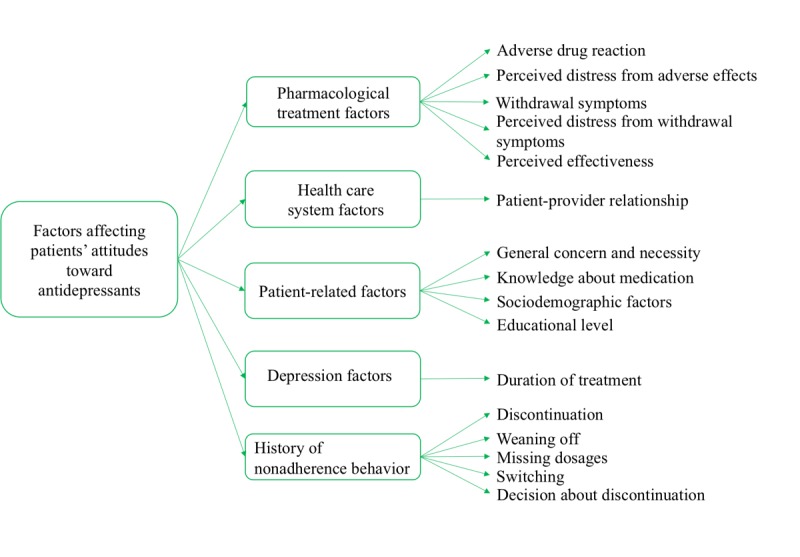
Final themes for the case study “attitude to antidepressants”.

Figure 5 shows the final themes after refining them for the study *attitude toward antidepressants*. The refined themes were used for constructing the final analytical framework. Multimedia Appendix 10 presents the final analytical framework with all the themes and examples from patients’ drug reviews.

### Phase IV: Analysis of Findings

Before researchers summarize and interpret the data, they should evaluate the quality of the produced structured data. As assigning a certain observation of patient narratives to themes is a subjective process, a disagreement may happen between annotators (coders). In this section, we explain measures for computing IAA and then discuss summarizing and interpretation of the findings.

#### Computing Interannotator Agreement

Cohen kappa is the most popular method for computing IAA. It measures the agreement between 2 annotators who annotate N items (eg, 100 sentences) into M mutually exclusive themes (eg, 10 themes) and corrects the result for the agreement that would be expected by chance [40,41]. Possible values for the kappa coefficient ranges from –1 to 1, where 1 indicates complete agreement, 0 indicates completely random agreement, and –1 indicates complete disagreement. For the formula of Cohen kappa and detailed interpretation on the obtained values, see Multimedia Appendix 11 [42,43].

To improve the quality of produced structured data, researchers may decide to annotate each document (eg, patient post) by more than 2 annotators. In this case, Fleiss kappa (an adaptation of Cohen kappa for 3 or more raters) should be used for computing the IAA [41].

There are other methods for calculating IAA, such as pairwise agreement. If the annotation task requires identifying terms or phrases and determining their correct boundaries (eg, identifying sign or a symptom) in the patient’s posts, the pairwise agreement would be an appropriate measure. The kappa coefficient would not be suitable because the chance agreement is effectively 0 in this case. Please see Zolnoori et al [7] for the formula and examples related to computing pairwise agreement.

#### Interpretation of Findings

If the structured data developed during the process of content analysis is rich enough, it can provide substantial insight into patients’ concerns, needs, preferences, and attitudes. Interpretation of the result could start with a general description of the themes, followed by reporting the most frequent and infrequent identified patterns, and finally reporting the unexpected patterns in data. Multimedia Appendix 12 provides a descriptive interpretation of the findings of the case study *access to diabetes medications.*

#### Implication of Findings

The findings of content analysis can go beyond a simple description of themes. In fact, it can be used for describing patterns or regularities, generating and testing hypotheses, describing a phenomenon and the associated factors, identifying problematic areas in the health care systems, or even developing predictive models to predict a specific patient’s behavior, such as medication nonadherence behavior. For example, for the project *attitude to antidepressants*, we tested the association between themes (variables) and patient attitudes toward antidepressants using statistical methods (analysis of variance and chi-square test) [11].

## Discussion

### Efficient Qualitative Research

Qualitative content analysis approaches are nonlinear, and iterative processes are more complicated than quantitative approaches because they are less structured and standardized. There are no single guidelines for content analysis. Selecting a specific approach strongly depends on the aim of the study, the research question, and the type of qualitative data. Researchers collecting and analyzing qualitative data, such as patient narratives, often wish to have a systematic approach including the detailed instruction on how to conduct qualitative research efficiently.

This study provided a systematic framework for the content analysis of patient-generated narratives in online health forums (communities). The systematic framework was built on text mining approaches for data collection and data preprocessing and qualitative content analysis using the framework method with the deductive-inductive approach for themes generation. We showed the feasibility and usefulness of the proposed systematic framework using 2 case studies: (1) a published study with a focus on identifying factors affecting patients’ attitudes toward antidepressants [11] and (2) an ongoing study with the focus on strategies and solutions of patients with diabetes facing financial difficulties to access medications and supplies. The data sources for these 2 studies were from online health forums. Using the systematic framework specified in this study, we could generate high-quality structured data that not only could identify the different dimensions and aspects of patients’ experiences but also could be used for testing hypotheses concerning the relationships between variables (themes) [11]. In addition, the structured data could also be used to train text mining algorithms to identify specific health-related information from patients’ narrative data [7].

The core component of the proposed framework (phase 3) is the framework method for qualitative content analysis [10]. Its prominent feature is the facilitation of *constant comparative method* through a matrix structure of the data. The matrix structure provides an intuitively structured overview of the summarized data that can facilitate and accelerate the identification of patterns and themes by highlighting the contradictory data and irregular cases. More importantly, it keeps a clear map between original data and themes in the analytical framework, indicating illustrative quotes for themes.

We used a combination of deductive-inductive approach to develop themes for both case studies in this study. However, the proposed systematic framework can be applied equally to studies aimed to use only inductive or deductive approach for data analysis. Our literature review showed that studies with focus on the qualitative content analysis of patient narratives in online health forums mostly used inductive approach for theme generation. But, we showed that the deductive analysis could accelerate the inductive analysis of patient narratives and identify new patterns and themes. There are major differences between this systematic framework proposed in this study and the framework of content analysis suggested by other papers. Please see Multimedia Appendix 13 for this difference [44,45].

### Limitations

We acknowledge some limitations with our proposed framework:

1. It is not appropriate for the analysis of very heterogeneous patients’ narratives, for example, if the patients’ experiences and the discussions in health forums are very diverse and cover a wide range of health topics. The systematic framework is most suitable for the studies with research questions targeting specific patient cohort with shared health concerns and experiences (eg, medications’ effects or difficulty in access to a health services).
2. It is not suitable for qualitative studies aiming at developing a theory or analysis of the structure of the experiences or language or the social context associated with the language. The research team may adopt other qualitative approaches to achieve the aims, such as approaches for developing theories derived from the data (eg, grounded theory) [46], approaches with focus on identifying the structure of an individual’s experience (eg, phenomenology) [47], approaches with concern about the relationship between language and the social structure in which the language is used (eg, discourse analysis and ethnomethodology) [48], and approaches designed with the aim of investigating intention and language (eg, narrative analysis) [49].
3. Although it provides a detailed instruction on analysis of patient posts, which may save time and resources similar to other qualitative analysis methods, it is still time consuming and resource intensive when involving time needed for developing guidelines and training annotators. This time needs to be factored into the study methods and approach.

### Lessons Learned

Previous experiences with and lessons learned from the content analysis:

- Qualitative content analysis may seem confusing and complicated for novice researchers. They may find this process to be chaotic and grapple with the qualitative research terms and concepts, such as patterns, categories, and themes. But experiencing chaos during the analysis is normal. Qualitative researchers need to be open to the complexity of content analysis [50] and improve their experience for systematic thinking.

- During the content analysis process, it would be very helpful to review the research questions constantly. Frequently referring to the research question and aim of the study will help researchers to stay focused on only dimensions of the dataset that answer the research question. It is also very important to take a note of new ideas and identified themes during the whole process of analysis. If the data analysis is conducted in an Excel sheet, assigning a column to notes and ideas would be very useful.

- Content analysis is a very time-consuming process and unexpectedly challenging [12]. The research team should plan ahead to have sufficient time to think, reflect, and then conduct a review of the analysis.
- It is important to avoid any preunderstanding of the dataset to minimize the risk of bias during the process of content analysis and interpretation of the results [9].

- It is important to have a weekly meeting to discuss new ideas and identified patterns in the group. All team members should be open and receptive to new ideas. The research team should proceed with defining and updating analytical framework based on the summary of the meeting discussion each week.
- Creating a table or figure containing information about the process of analysis from the raw data to a meaningful unit of analysis, to the identified themes with examples from patients’ post would be very useful. Including the figure or table in the manuscript of the study will show the validity of the study and improve appreciation of reviewers and readers of the study’s findings.

### Conclusions

Exploring patient-reported experiences and concerns in online health care forums (communities) and translating such content into meaningful concepts (themes) has become a challenge for health care researchers and health care providers. In this study, we introduced a systematic framework as a rigorous and standardized method to collect patient-reported experiences from online forums and convert their content to themes that are reliable and easily interpretable. The framework was built on the text mining approaches and the framework method with the deductive-inductive approach that benefit both researchers and clinicians by minimizing the cost, time, and human errors during the process of data processing and analysis. We showed the reliability and efficiency of this framework using 2 case studies: one identifying factors associated with patients’ attitude toward antidepressants and the other identifying solutions and strategies of patients with diabetes facing financial difficulties to access medications and supplies. Finding meaningful information through such a process indicates authentic dimensions and aspects of patient experiences and sheds light on patients’ concerns, needs, preferences, and values, which are the core of patient-centered care.

## Appendix

Multimedia Appendix 1 Definition of HTML, API, Web crawler, Python package, and XML.

Multimedia Appendix 2 Kinship terminology and UMLS (Unified Medical Language System).

Multimedia Appendix 3 Example of a formula for determining the sample size.

Multimedia Appendix 4 Examples of regular expression codes for reducing noise patterns in patients' posts.

Multimedia Appendix 5 Case study #2: strategies and solution of diabetes patients with financial difficulties for accessing medications and supplies.

Multimedia Appendix 6 Operational definitions for all predefined themes for the case study “identifying factors affecting patients’ attitudes towards antidepressants”.

Multimedia Appendix 7 New themes generated through inductive approach for the case study “identifying factors affecting patients’ attitudes towards antidepressants”.

Multimedia Appendix 8 Rules for refining the identified themes.

Multimedia Appendix 9 Guidelines for annotating the patients comments for the case study “diabetes patients solutions to access medications and supplies in the context of financial difficulties”.

Multimedia Appendix 10 Final analytical framework for the case study “identifying the underlying factors affecting patient attitudes toward antidepressants”.

Multimedia Appendix 11 Interpretation of the obtained results from the Cohen kappa.

Multimedia Appendix 12 Descriptive interpretation of the findings of the case study “access to diabetes medications”.

Multimedia Appendix 13 The major difference between the systematic framework and other content analysis frameworks.

## Abbreviations

ADR: adverse drug reaction
API: application programming interface
IAA: interannotator agreement
IRB: institutional review board
NLP: natural language processing
UMLS: Unified Medical Language System
