# Supplementary material for: A Systematic Framework for Analyzing Patient-Generated Narrative Data: Protocol for a Content Analysis
Source: JMIR Res Protoc. 2019 Aug 26;8(8):e13914. doi: 10.2196/13914 (PMC6786846; doi:10.2196/13914)
Supplement: Multimedia Appendix 1 [file resprot_v8i8e13914_app1.pdf]

Multimedia Appendix 1: Definition of HTML, API, Web crawler, Python package, and XML

- HTML: “Hypertext Markup Language is the standard markup language for creating web pages and web applications” (https://en.wikipedia.org/wiki/HTML).

- API (Application Programming Interface), a set of functions and procedures allowing the applications to communicate directly with the website (healthcare forums), and collect the requested information from the healthcare forum and community. (https://en.wikipedia.org/wiki/Application_programming_interface)

- Web crawler: Web crawler is a web search engine that can systematically borrow the content of a website and collect the requested information.

- Python package: python is a high-level programming language (https://www.pythonforbeginners.com/learn-python/what-is-python/). Python package is a collection of modules in directories that give a package hierarchy. “A Python module is simply a Python source file, which can expose classes, functions and global variables.” (https://softwareengineering.stackexchange.com/questions/111871/module-vs-package)
  Please see the following link for more information: https://docs.python.org/3/tutorial/modules.html

- XML: “Extensible Markup Language (XML) is a markup language that defines a set of rules for encoding documents in a format that is both human-readable and machine-readable.” https://en.wikipedia.org/wiki/XML
