# Supplementary material for: A Systematic Framework for Analyzing Patient-Generated Narrative Data: Protocol for a Content Analysis
Source: JMIR Res Protoc. 2019 Aug 26;8(8):e13914. doi: 10.2196/13914 (PMC6786846; doi:10.2196/13914)
Supplement: Multimedia Appendix 2 [file resprot_v8i8e13914_app2.pdf]

Multimedia Appendix 2: Kinship terminology and UMLS (Unified Medical Language System)

Kinship terminology includes terms that patients' families use to refer to the patients they care for in their posts. Therefore, using this terminology as a content specific feature helps to differentiate patient posts their caregivers'. Medical terminology specified in the Unified Medical Language System (UMLS) can distinguish patients and caregivers' posts from clinicians. Clinicians mostly use medical standard vocabularies to discuss aspects of healthcare, patients use lay language and subjective terms to explain their experiences in their forums that cannot be identified using medical terminologies .
