# Supplementary material for: A Systematic Framework for Analyzing Patient-Generated Narrative Data: Protocol for a Content Analysis
Source: JMIR Res Protoc. 2019 Aug 26;8(8):e13914. doi: 10.2196/13914 (PMC6786846; doi:10.2196/13914)
Supplement: Multimedia Appendix 3 [file resprot_v8i8e13914_app3.pdf]

Multimedia Appendix 3: Example of a formula for determining the sample size

For example, in the study "attitude to antidepressants" we first stratified the retrieved data based on the patients' satisfactions with drugs (ranged from 1 to 5), and then we used the following formula to determine the sample size:

$$Sample\ Size = \frac{\frac{z^2 \times p(1-p)}{e^2}}{1 + (\frac{z^2 \times p\ (1-p)}{e^2\ N})}$$

- "Z-score" (z): The Z-score describes how many standard deviations a given measurement lies above or below a size of population mean for a specific parameters [1]. The value for Z-score is usually picked up from the following list: [1.28, 1.44, 1.65, 1.96, 2.58] which is equivalent to the following confidence interval [80%, 85%, 90%, 95%, 99%]. For the study "attitude towards antidepressants," we chose the value of 1.65 (for confidence interval 90%), which is a common confidence interval to have a sample representative of population. Increasing the value of Z-score increases the size of sample, and in turn the power of the study [2]. However, regarding the time and cost associated with the process of content analysis, we decided to set the confidence interval on 90% to have a reasonable sample size regarding our available time and financial sources.
- Margin of error (e): A percentage that shows to what extend the sample reflects the view of overall population. The common values for "e" are 1%, 5%, and 10%. The smaller the margin of error, the obtained results are closer to the exact values of population [2]. We set the value on 5%, which is a common value for Margin of error.
- P (standard deviation): P is the standard deviation. If we did not have any information about standard deviation, we set it on 50%, which gives the largest sample size.
- N is population size: in online healthcare forums, N is the number of posts or discussion threads.

1. Curtis AE, Smith TA, Ziganshin BA, Elefteriades JA. The mystery of the Z-score. Aorta. 2016;4(04):124-30.
2. SurveyMonkey. Sample size calculator. 2019 [May 2019]; Available from: https://www.surveymonkey.com/mp/sample-size-calculator/.
