# Supplementary material for: A Systematic Framework for Analyzing Patient-Generated Narrative Data: Protocol for a Content Analysis
Source: JMIR Res Protoc. 2019 Aug 26;8(8):e13914. doi: 10.2196/13914 (PMC6786846; doi:10.2196/13914)
Supplement: Multimedia Appendix 4 [file resprot_v8i8e13914_app4.pdf]

Multimedia Appendix 4: Examples of Regular Expression Codes for Reducing Noise Patterns in the Patients Posts for Drug Reviews for Case Study “Identifying Factors Affecting Patients’ Attitudes towards Antidepressants”

Source: Zolnoori, M. (2017). “Utilizing Consumer Health Posts for Pharmacovigilance: Identifying Underlying Factors Associated with Patients’ Attitudes Towards Antidepressants.” Theses and Dissertations. 1733. https://dc.uwm.edu/etd/1733

<table>
<tr><th>Noisy patterns</th><th>Example</th><th>Regular expression code</th></tr>
<tr><td>Inserting space after full stop punctuations and the beginning of a new sentence</td><td>“I have suffered many side effects from the prednisone used to treat the symptoms. I am just now starting to feel better.”</td><td>comments = re.sub (r'([a-zA-Z])([\.?!])([a-zA-Z])',r'\1\2 \3', comments)</td></tr>
<tr><td>Removing Emails</td><td>com.uk@hotmail.comsevere</td><td></td></tr>
<tr><td>Replacing dot before parenthesis with space</td><td>“But about a year later I tried others and could tolerate them much better. (Such as Lexapro and Prozac.). I hate it.”</td><td>Comment = re.sub(r'\.(\s*?\([^)]*\)\s*\.)', r'\1', Comment)</td></tr>
<tr><td>Removing repeated punctuations from the end of the sentence</td><td>“Think menopause is a huge factor in this....i have only been on generic zoloft for about a week and a half”</td><td>comment = re.sub (r'([,.;:!?-])\1+', r'\1',comment)</td></tr>
<tr><td>Replacing “.” with “,” from the list of side-effects (ADRs)</td><td>“Night terrors. Vivid dreams. Nightmares. auditory hallucinations everyday!!!!! never take this drug!!!”</td><td>comment = re.sub(r'\.(\s*(?!(?:i|she|he|He|She|I|we|We|They|they|it|It|You|you)\b)\w+(?:\s+\w+)?\s*)(?=[^\w\s]|$)', r',\1', comment , flags=re.I)</td></tr>
<tr><td>Replacing hyphen with the dot when hyphen indicates the full stop of the sentence</td><td>“Severe teeth clenching/TMJ which caused excruciating headaches - clenching came on right after taking the dose and would gradually wear off”</td><td>Comment = re.sub (r'(\s+)(\-)(\s+)', r'\1.\3 ', comment)</td></tr>
<tr><td>Replacing the comma with the dot, when comma indicates full stop of a sentence</td><td>“Effexor People are still having problems with this medication even after being off of it for 8 months or more, please don't take this medication.”</td><td>Comment = re.sub (r'(\s+)(\,)(\s+)', r'\1.\3 ', comment)</td></tr>
<tr><td>Replacing dot after numbers when numbers indicate the order of items in a list</td><td>“After two to three hours vomit 2. Stiff muscles (Lack of oxygen?) 3. Spacey, out of sync with time (as in Where did that tree come from? What am I doing?) 4. Weird, (as in How did that tree get there?). 5. Weak muscles”</td><td>comment = re.sub(r'(\d)\.(\s?[a-zA-Z])',r'\1) \2' ,comment)</td></tr>
<tr><td>Inserting space between parenthesis and the following words</td><td>"Was deadly sick for 2 wks and he kept me on it regardless, upped the dosage three times over a year because it wasn't working(duh, it was my thyroid!)That dr. is history, btw! This time, I was very leary of taking anything."</td><td>comment = re.sub(r'(\))(\w+)',r'\1 \2' ,comment)</td></tr>
<tr><td>Replacing (, - ,) before “overall” with dot</td><td>and I was only on 2,5 months - overall - DOES do what it is supposed to do - you need to</td><td>comment= re.sub(r'[,-](\s*[oO]verall?\s+)',r'. \1' ,comment)</td></tr>
</table>

	weight whether it is worth the side effects.	
Patients did not use any punctuations indicating sentence stop	I was hoping it would eventually go away so I continued to take it and after the 6th day of the same intense side effects I finally had to just chunk them in the trash.	No solution
Incomplete sentences	i recommend the cookies.. PS.. i have not gained any weight.. and if	No solution
