# Supplementary material for: A Systematic Framework for Analyzing Patient-Generated Narrative Data: Protocol for a Content Analysis
Source: JMIR Res Protoc. 2019 Aug 26;8(8):e13914. doi: 10.2196/13914 (PMC6786846; doi:10.2196/13914)
Supplement: Multimedia Appendix 5 [file resprot_v8i8e13914_app5.pdf]

Multimedia Appendix 5: Case Study 2 - Strategies and solution of diabetes patients with financial difficulties for accessing to medications and supplies

To generate themes for the study “access to diabetes medications”, we followed the same procedure used for generating themes for the project “attitude towards antidepressants.” Here, we explain briefly the themes generated in each step of the procedure for this study.

Generating themes using deductive analysis: Our literature review showed that studies with the focus on cost related non-adherence in diabetes patients with financial difficulties identified a set of solutions and strategies that the patients use to access their medications and supplies. For example, findings of the literature showed that applying for assistantship programs from public organizations or asking the physicians for less expensive medications are some of the patients’ strategies to access medications. The studies mostly used interview and questionnaire methods to collect data from patients. However, patient posts in forums may include information that was not identified by the study in the literature.

In the step of deductive analysis of self-claimed diabetes patient posts with financial difficulties, we used the identified strategies and solutions from the literature as pre-defined themes (**Figure 1**) for developing the initial analytical framework. To develop the initial analytical framework, we used the same procedure explained in section “phase III: content analysis” for developing the initial analytical framework in the study “attitude to antidepressants.” We developed guidelines and operational definitions for the pre-defined themes before coding the posts using the initial analytical framework.

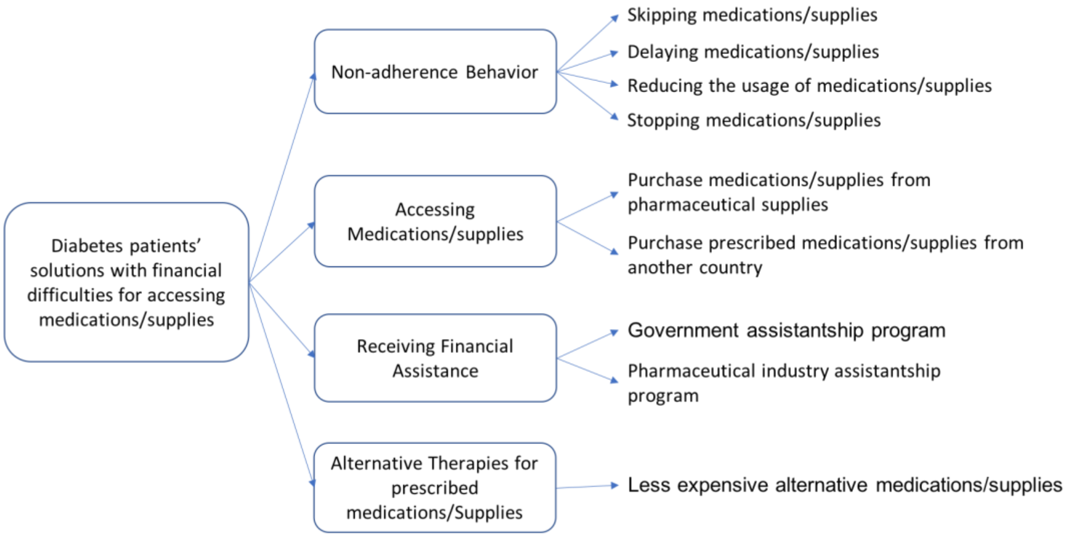

Appendix D-Figure 1. Generated themes using deductive analysis for the case study “access to diabetes medications”

Generating themes using inductive analysis: Diabetes Patients with financial difficulties may report other strategies and solutions for accessing to medications and supplies in online forums that cannot be identified using conventional data collection methods. Therefore, we used inductive analysis to generate new themes for the meaningful segments of patient posts that could not fit into the pre-defined themes in the initial analytical framework. **Figure 2** shows new themes developed through inductive analysis for the study “access to diabetes medications.”

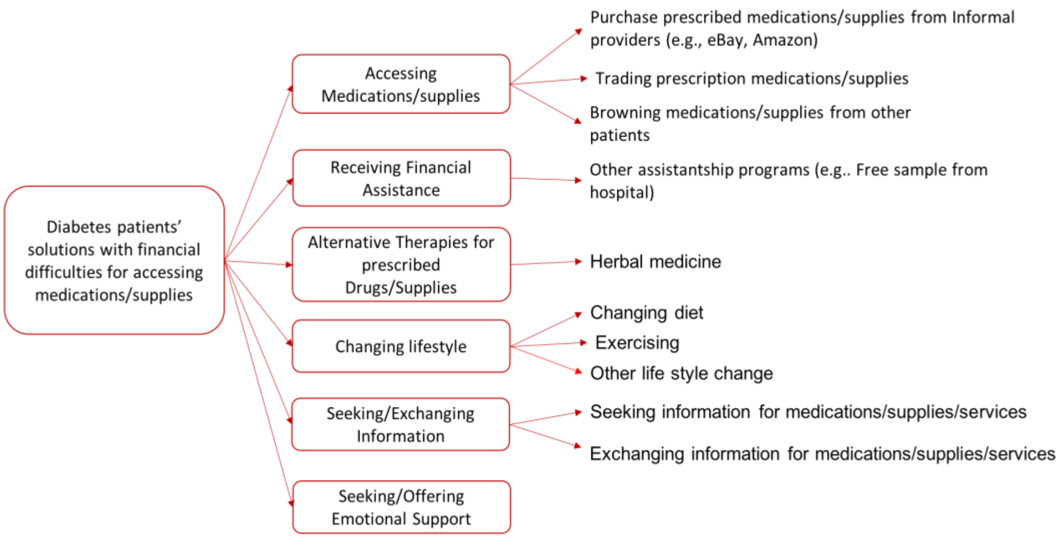

Figure 2. Generated themes using inductive analysis for the case study "access to diabetes medications"

Refining themes and developing the final analytical Framework: Similar to the project "attitude to antidepressants," we refined the themes generated through deductive and inductive analysis by creating a set of rules. In contrast with the study "attitude to antidepressants", we did not set any threshold on the number of posts that should be assigned to a theme, because discovering any rare strategy or solution that diabetes patients used to access medications or supplies were important for the purpose of this study. However, similar to the study "attitude to antidepressants," we set some rules to combine themes having overlaps in the definition or subjectively it is difficult to distinguish between them.

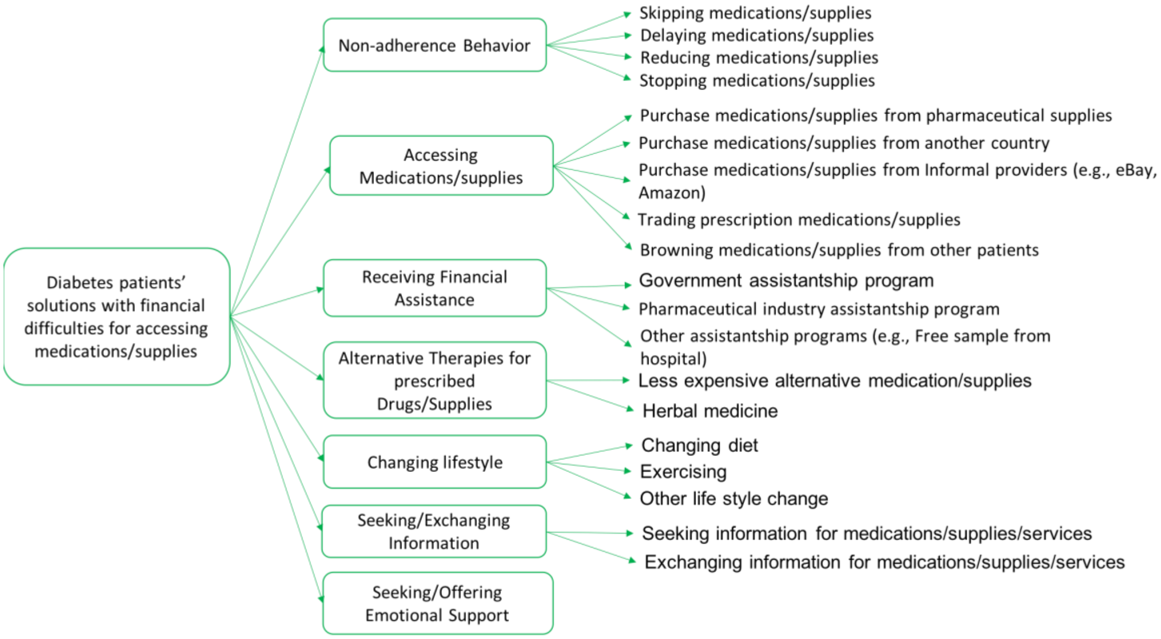

Figure 3. Final themes for the case study "access to diabetes medication"

Figure 3 shows the final themes produced through inductive and deductive analysis for the study "access to diabetes medications."
