# Supplementary material for: A Systematic Framework for Analyzing Patient-Generated Narrative Data: Protocol for a Content Analysis
Source: JMIR Res Protoc. 2019 Aug 26;8(8):e13914. doi: 10.2196/13914 (PMC6786846; doi:10.2196/13914)
Supplement: Multimedia Appendix 6 [file resprot_v8i8e13914_app6.pdf]

Multimedia Appendix 6

Operational Definitions for all Pre-Defined Themes for the Case Study “Identifying Factors Affecting Patients’ Attitudes towards Antidepressants”

Source: Zolnoori, M. (2017). “Utilizing Consumer Health Posts for Pharmacovigilance: Identifying Underlying Factors Associated with Patients’ Attitudes Towards Antidepressants.” Theses and Dissertations. 1733. https://dc.uwm.edu/etd/1733

Please see Table 1 and Table 2.

Table 1: Description of Predefined Themes Developed Deductively from the Literature

Categories	Factors (predefined codes)	Description
Pharmacological treatment factors	Perceived effectiveness	The patient’s subjective assessment of antidepressant helpfulness in the reduction of depression symptoms, enhancing emotional and cognitive functionalities, and in overall, enhancing life quality.
	Perceived necessity	The patient’s subjective assessment of antidepressant necessity in improving and maintaining current and future health conditions. For example, the patient may find an antidepressant vital in reducing the risk of relapse.
	Perceived concern	The patient’s subjective assessment of antidepressants harmful effects in long-term. The patient may view antidepressant as an agent, which is addictive, take control over feelings and thought, and altering personality in long-term.
	Side-effects	Any adverse reactions that the patient reports as adverse reactions to antidepressants intake. Antidepressants’ adverse reactions may include physiological side-effects, emotional syndromes, cognitive impairment, and limitations on daily functioning, and in overall reducing quality of life.
	Perceived distress from side-effects	The patient’s perceived distress from antidepressants side effects depends on patient’s self-attention to the internal bodily sensation that may have an influence on patient tolerability of side effects. The patient may show the distress by using adjective showing severity of the side effects, negative impact on work or daily activities, or visiting emergency department. A severe side-effect including self-harm and suicidal ideation or attempt also reflects high-perceived distress.
Healthcare system factors	Patient-provider relationship	The patient expresses their satisfaction or dissatisfaction with healthcare providers from various aspects, such as perceived support from providers or perception of healthcare providers’ knowledge about illness and treatment.
	Healthcare settings	The patient may demonstrate a higher level of trust and satisfaction towards diagnosis and treatment offered in a psychiatric setting compared with a primary care setting.
	Affordability	The patient may complain about the high cost of antidepressants and the lack of insurance coverage.
Psycho-social factors	Stigma and cultural related factors	The patient may express their concerns about stigma and cultural-related factors that may make the prolonged pharmacological treatment notoriously difficult for patients. For example, the public may view antidepressants intake as a sign of weakness or incapacity to deal with daily emotional distress that may influence on patient acceptance of antidepressants.
	Partner support	The patient may express their perceived support from partners (family and friends) about having depression as proper diagnosis and having an antidepressant as proper treatment.
Patient-related factors	General concern and necessity	The patient may express their general view towards medications. For example, they may view all the medications harmful and believe natural remedies and changing lifestyle will have a better healthcare outcome than pharmacological treatment.

	Knowledge about medication	The patient may complain about lack of knowledge of side effects or complexity of treatment, the proper time of discontinuation, and the withdrawal adverse effects.
	Education level	The patient may disclose their level of education in the comments or the section of demographic information.
Disorder related factors	Patient insight about source of depression	The patient may express their insight towards the source of depression, and severity of symptoms. For example, the patient may attribute the source of depression to the psychological problems rather than to biological factors.
	Severity of depression /symptoms	The patient with major depression may have a higher tolerability of adverse effects. Also, patient's perceived effectiveness of antidepressant may be higher in the patients.
	Type of depression	Type of depression may be an important factor in shaping patient's attitude toward antidepressants.

Table 2: Predefined Themes Developed Deductively with Examples from Patients' Posts for Drug Reviews

Predefined codes	Sub-codes	Examples from patients' reviews
Perceived effectiveness	Positive	"Anyway, my life is on track, I have nothing to be depressed or sad about."
	Negative	"But I still haven't noticed any change in my energy or anxiety."
Perceived necessity	Positive	"All in all, I love it. I have not had a depressed moment since I've been on it, approx. 8 month. I do not think I cannot live without it."
	Negative	"There's no doubt that Effexor XR saved my life, however long-term use is not the best."
Perceived concern	Concern about side-effects	"Pure poison!"<br>"I was so scared to take it that I delayed it because I read all the comments on this site."
	Concern about withdrawal symptoms	"I am worried about trying to cut down gradually as I read so much scary stuff about going off it."<br>"The withdrawal is hell. You would think you were going through heroin withdrawals."
ADRs		"Typical with effexor XR- Dizzy, jaw tight, teeth grinding."
Perceived distress from side-effects	Tolerable (low)	"I had tolerable, minimal side effects."<br>"I didn't really experience any side effects..."<br>"The side effects are far less hassle than being in my dark depression."
	Intolerable (high)	"The side effects are hell."<br>"No wonder I suffered so many side effects."
Physician patient - interaction	Positive	"My pharmacist convinced me that it was safe for me to use along with my topamax, and encouraged me to try it. I'm really glad I did."
	Negative	"I am suffering and should of not trusted the NP who prescribed the pills without first checking it out."
Healthcare setting *		"Respondents did not explicitly mention in which healthcare setting they received treatment for depression."
Affordability *		"If the depression doesn't eventually cripple you, the costs will."<br>"It is too expensive if you don't have insurance!"
Stigma and cultural related factors		Non of the respondents did not explicitly complain about stigma or did not present any information indicating cultural factors affecting on patients attitudes antidepressants.
Partner support*	Positive	No information is available.
	Negative	"My husband has no compassion to my withdrawal symptoms."
General concern and necessity*		"Do NOT believe the MDs and pharm corp. hype and lies! FLEE!"
Demographic information		"Basic demographic information including age and gender was reported by respondents."
Knowledge about medication		"No one ever told me even as I was seeing my psych and psychologists weekly, and my physician, that what I was experiencing could be from the drug."
Education level*		Patient did not disclose their education level in their comments. The website also did not provide a field to request responded educational level.
Patient insight about depression *	Positive insight	"Mental illness (i.e. lack of serotonin) is just like any other defect the human body can have."
	Negative insight	No information is available.
Severity of depression/symptom		Some of the respondents in the field of "reason of prescription" or their comments mentioned severity of depression or their symptoms, such as:<br>"The depression is so terrible that I am very keen to give Lexapro a fair try."
Type of depression		Some of the respondents mentioned type of their depression in the field of "reason of prescription" or in the comments.
Duration of depression		Most of the respondents in the filed of "duration/dosage" reported duration of antidepressant's usage.
