# Supplementary material for: A Systematic Framework for Analyzing Patient-Generated Narrative Data: Protocol for a Content Analysis
Source: JMIR Res Protoc. 2019 Aug 26;8(8):e13914. doi: 10.2196/13914 (PMC6786846; doi:10.2196/13914)
Supplement: Multimedia Appendix 7 [file resprot_v8i8e13914_app7.pdf]

Multimedia Appendix 7

New Themes Generated Through Inductive Approach (Open Coding) for the Case Study "Identifying Factors Affecting Patients' Attitudes towards Antidepressants"

Source: Zolnoori, M. (2017). "Utilizing Consumer Health Posts for Pharmacovigilance: Identifying Underlying Factors Associated with Patients' Attitudes Towards Antidepressants." Theses and Dissertations. 1733. https://dc.uwm.edu/etd/1733

New Themes	Sub-Themes	Examples
Withdrawal symptom		"I weaned slowly from 150mg to 75 to 37.5 and off. I feel nauseous a lot and my depression and social anxiety has returned almost 100%."<br>"Do not wean off Effexor too soon as i had one very bad day and experienced mild hallucinations."
Perceived distress from withdrawal Symptoms	Low (tolerable)	"Could miss a dose or two and no problem."
	High (intolerable)	"Trying to come off of the medication is very difficult."
Overall attitude	Positive	"I think that this drug is great, but only for certain people."
	Negative	"This drug is just a poison."<br>"This drug ruined my life."
Drug indication		"My depression mostly manifested in an inability to start new projects, rather than any feelings of sadness."<br>"I used to be so depressed and anxious, that I didn't want to leave the house. I have a long history of depression and anxiety."
Recommendation to others	Positive	"I would definitely recommend Effexor XR."
	Negative	"Do not take this medication!!!!"
Experience of withdrawal	Discontinuation	"I stopped this drug after two days."
	Weaning off	"Cutting the dose in half, when I went down to taking none, experience withdrawal effects."
	Missing dosages	"Cannot miss a single dose or I feel awful."<br>"I experience when missing a dose."
	Switching	"I have insisted on stopping the Effexor, and now the doctor is pushing pristiq (the "new and improved" Effexor)."<br>Switched to Luvox which I am finding much more beneficial."
	Decision about discontinuation	"I think I'm going to quit."
Dosage/duration		"I started out on Effexor XR 75 mg, and was slowly raised from there to Effexor XR 300 mg. Then I was changed to the generic Venlafaxine XR 300 mg."
Experience with other medications		"Started taking it after Prozac and (can't remember the name) made my stomach hurt so badly."
Problem with financial support		"I did a rapid decline on my Cymbalta because I lost my insurance."
Problem with social support		"Thankfully I have a wonderful husband who helped me past the side-effects."
Not applicable		"Hope the comments help and good luck."

	“I honestly can't tell a difference when I am on or off of Effexor XR.”
