# Supplementary material for: A Systematic Framework for Analyzing Patient-Generated Narrative Data: Protocol for a Content Analysis
Source: JMIR Res Protoc. 2019 Aug 26;8(8):e13914. doi: 10.2196/13914 (PMC6786846; doi:10.2196/13914)
Supplement: Multimedia Appendix 8 [file resprot_v8i8e13914_app8.pdf]

Multimedia Appendix 8: Rules for refining the identified themes
Case Study: "Identifying Factors Affecting Patients' Attitudes towards Antidepressants"

Source: Zolnoori, M. (2017). "Utilizing Consumer Health Posts for Pharmacovigilance: Identifying Underlying Factors Associated with Patients' Attitudes Towards Antidepressants." Theses and Dissertations. 1733. https://dc.uwm.edu/etd/1733

1) Each theme in the predefined framework that did not fit with the information provided in reviews was excluded from the final framework. For example, we removed "healthcare setting," "stigma and cultural related factors," "educational level," and "dosage of medication."
2) If a predefined or a new theme fit in less than 5% of information provided in reviews, we excluded them from or merged them with other themes in the final framework. Similarly, "affordability," "partner support," and "patient insight about depression" were excluded and "general concern and necessity" was merged with "**overall attitude".**
3) All themes that were conceptually related and subjectively difficult to distinguish between their applicability to a segment of text were combined in the final framework. Accordingly, "perceived necessity" and "perceived effectiveness" are conceptually related and subjectively difficult to distinguish in applying to segments of text, therefore they were combined in the final framework as "perceived effectiveness". For example, the following segment can be labeled as both "perceived effectiveness" and "perceived necessity".

"All in all, I love it. I have not had a depressed moment since I've been on it, approx. 8 month".

4) Although ADRs and withdrawal symptoms are conceptually related and both reflect adverse drug reaction, we distinguished between them as two separate themes in the final framework. A patient may receive minimal distress from an antidepressant's ADRs, but perceive severe withdrawal symptoms during the process of discontinuation, which may negatively affect the patient's attitude. Distinguishing ADRs from the withdrawal symptoms is important for conducting clinical trials and designing interventions aimed to help patients manage the process of antidepressants' discontinuation.
5) Perceived concern for the prescribed drug includes segments of text in which patients expressed their perceived distress from ADRs, WDs, or the overall distress they received from the drug. Thus, we defined two new themes "perceived distress from ADRs" and "perceived distress from WDs" to express perceived express towards ADRs and WDs. For the segment of text in which a patient expressed overall perceived distress from the drug without distinguishing between WDs and ADRs, we assign it to the theme "overall attitude".
6) Some patients directly mentioned how the information obtained from the online sources including online messaging boards influenced their attitude toward antidepressants. This information can provide important evidence about the impact of online sources on shaping attitudes toward medications. However, because of the paucity of this information in the sample for this study, we did not define any new theme for this expression. Here are examples of such expression:
   - *"I don't want all those withdrawals I m reading about."*
   - *"I was so scared to take it that I delayed it because I read all the comments on this site."*
