# Supplementary material for: A Systematic Framework for Analyzing Patient-Generated Narrative Data: Protocol for a Content Analysis
Source: JMIR Res Protoc. 2019 Aug 26;8(8):e13914. doi: 10.2196/13914 (PMC6786846; doi:10.2196/13914)
Supplement: Multimedia Appendix 9 [file resprot_v8i8e13914_app9.pdf]

Multimedia Appendix 9

Guidelines for Annotating the Patients Comments using the Identified Themes for the case study "Diabetes Patients Solutions to Access Medications and Supplies in the Context of Financial Difficulties"

The following table provides a summary of the identified themes for the content analysis of diabetes patients' comments with financial difficulties.

Identified Themes for Content Analysis of Diabetes Patients' Posts with Financial Difficulties

Themes	Sub-themes
Discussing Insurance Choices or Insurance Coverage for a Specific Drug/Device/Supply	
Alternative Therapies for Prescription Drugs, Devices, or Supplies	Using less expensive pharmaceuticals drugs/devices/supplies<br>Using herbal medicine
Improving Diabetes Management by Changing Lifestyles	Changing diet<br>Exercising<br>Quitting smoking or drinking<br>Other life style changes (e.g., I've made a decision to take care of myself)
Accessing Prescription Drugs, Device, or Supplies	Buying prescription drugs/devices/supplies from informal providers (e.g., eBay, amazon)<br>Buying prescription drugs/devices/supplies from formal providers<br>Trading for prescription drugs/devices/supplies<br>Bought prescription drugs/devices/supplies from another country<br>Borrowed/buying drugs/devices/supplies from others
Receiving Financial Assistance	Applying/receiving for financial assistance programs from government<br>Applying/receiving for financial assistance program from pharmaceutical companies<br>Applying/Receiving other assistance programs (e.g., requesting some free samples from providers)
Discussing Non-adherence Behavior to Reduce Cost	Reducing the usage of the prescription drugs/devices/supplies<br>Skipping prescription drugs/devices/supplies<br>Delay refilling the prescription drugs/devices/supplies filled<br>Stopped usage of the prescription drugs/devices/supplies due to cost
Exchanging/Seeking Information	Seeking information for drugs/devices/supplies<br>Seeking information for healthcare services<br>Exchanging information for drugs/devices/supplies<br>Exchanging information for healthcare services
Seeking/Offering	

Emotional Support	
Others	

1. Discussing Insurance Choices or Insurance Coverage for a Specific Drug/Device/Supply

Definition: Patients may explicitly or implicitly state their insurance status (they have insurance or not) or coverage of the insurance for a Drug/Device/Supply.

Examples:

- "I had cigna and I swear it felt like they did everything they possibly could to make anyone who has diabetes financially suffer". (*cigna is a type of insurance and the patient is complaining about type of services provided by the insurance*)
- "Waiting to hear back from Medicaid so no insurance right now". (The *patient stated his/her insurance status here)*.
- "My insurance covered a brand new animas pump with the built in CGM". (The *patient stated the insurance coverage for a medical supply here*).

2. Alternative Therapies for Prescription Drugs, Devices, or Supplier

2.1 Using less expensive pharmaceuticals drugs/devices/supplies

Definition: Patients may explicitly or implicitly state that they plan to replace or they replaced the prescribed drug/supply/device with the less expensive ones.

Examples:

- "My goal is to wean myself off insulin, subsisting on the low-cost metformin". (*The patient explicitly mentioned that they are going to substitute the insulin with low-cost metformin*).
- "In the 80's, they started pushing the humilin, which was a lot better.... Now I consider myself an old-timer, still using the NPH... But since in many poorer countries, all they can afford is NPH, I do not see them getting rid of NPH anytime soon, since the newer insulin's keep going up, up, up, in price, at least in the USA". (The *patient uses the NPH (a type of Pork insulin) instead of human insulin that is more expensive than pork insulin*).

2.2 Using herbal medicine

Definition: Patients may explicitly or implicitly mention that they replaced prescribed drugs with herbal treatment.

3. Improving Diabetes Management by Changing Lifestyles

3.1 Changing diet

Definition: Patients may explicitly or implicitly state that they changed or they plan to change their diet (e.g., using a low carb diet) to have a better control on diabetes symptoms (e.g., blood sugar) or to manage weight. Patients may also suggest changing diet as a solution for controlling diabetes symptoms or weight management to other patients in the forum.

Examples:

- "Last A1C was low for 10 days on a LC diet (20 or less carbs per day), my BG is running low 120s and below using 15 of Humalog BID." (*The patient used LC diet to have a better control on weight*).
- "Went LCHF a year ago and dropped it down to like 70-80 units per day."
- "I am not a big meat or fat eater, so on LC I have difficulty getting enough calories in to maintain my 115 pounds."

3.2 Exercising

Definition: If patients explicitly or implicitly stated that they could manage diabetes symptoms by exercising. Patients may also suggest to other patients to use exercising as a means for controlling of diabetes symptoms.

Examples:

- "I can go all out as fast as I can to where I breathe hard and its hard to talk (this is the aerobic exercise that really sucks up the sugar out of the blood).I always said that exercise never did anything immediate for my blood sugar, but I never did it to where I was breathing hard." (*The Patient implicitly here mentioned that they used exercising as means to control diabetes symptoms*).

3.3 Quitting smoking or drinking

Definition: If patients implicitly or explicitly mentioned or suggested quitting smoking or drinking can help to have a better control on diabetes symptoms.

3.4 Other life style changes (e.g., I've made a decision to take care of myself)

Definition: if patients implicitly or explicitly stated that they changed their life style to have a better control on diabetes symptoms. Patients may also suggest others to use life style changes to control diabetes.

Examples:

- "I'm good till next year, but I decided to make some radical diet lifestyle changes and see what happened."

4. Accessing Prescription Drugs, Device, or Supplies

4.1 Buying prescription drugs/devices/supplies from informal providers (e.g., eBay, amazon)

Definition: Patients may explicitly or implicitly state that they bought a drug/supply/device from informal healthcare settings (online or physical store) that their primary functions is Not dispensing drugs/ devices/supplies. Patients may also suggest other patients such stores to reduce cost of treatment.

Examples:

- "Its the strips that are costly, if I had to pay for mine it would be the Walmart Relion Prime meter many here use it." (*Primary function of Walmart is not dispensing drugs/supplies/devices.*)
- "I bought my own meter and strips on Amazon because they were much cheaper than using my insurance" (*Primary function of Amazon is not dispensing drugs/supplies/devices)*.

4.2 Buying prescription drugs/devices/supplies from formal providers.

Definition: Patients may explicitly or implicitly state that they purchased the drugs/devices/supplies from pharmacies that their primary functions is dispensing drugs/devices/supplies.

Examples:

- "I usually buy my medications from the pharmacy at the clinic."

4.3 Trading for prescription drugs/devices/supplies

Definition: Patients may explicitly or implicitly state that they would like to trade the drugs/devices/supplies for their other medical needs (other type of drugs/devices/supplies).

Examples:

- "Could pay a little or have basic lancets I could trade." (*In this sentence, the patient would like to try the lancets (a type of Insulin) for other treatment needs*)
- "Hi everyone, any advice on how to determine trade of supplies?"

4.4 Bought prescription drugs/devices/supplies from another country

Definition: Patients may explicitly or implicitly state that they bought or attempted to buy the drugs/devices/supplies from other countries because of lower prices.

Examples:

- "While there I used a courier service which had a mailing address in Miami then couriered the pump down from Guatemala."

4.5 Borrowing/buying/requesting drugs/devices/supplies from others

Definition: Patients may implicitly or explicitly request other participants in the forum to lend or sell drugs/devices/supplies. Some patients may also may implicitly or explicitly request free drugs/devices/supplies.

Examples:

- "Don't have insurance or a full time job so free or very inexpensive is preferable!"
- "If you're not in the area I can cover shipping but cannot afford to pay very much right now."

5. Receiving Financial Assistance

5.1 Applying/receiving for financial assistance programs from government/public health organizations

Definition: Patients may explicitly or implicitly state that they are receiving (received) financial assistance or they applied or a plan to apply for financial assistance from the government or public health organizations, such as the American Diabetes Association (ADA).

Examples:

- "I would apply for government assistance except I make too much, yet not enough to pay for the monthly cost that is equivalent to my mortgage."

5.2 Applying/receiving for financial assistance program from pharmaceutical companies

Definition: Patients may explicitly or implicitly mention that they are receiving (received), applied, or a plan to apply for financial assistance from pharmaceutical companies.

Examples:

- “apidra had a free program and lantus was 25 % off on a pump, so use Humalog only now.” (*Apidra iss a drug company)*.
- “I have a accu-check that i got for $20 on sale at rite aid and i get my strips free thru the company because i have no insurance.” (*accu-chek is a drug company*.)

5.3 Applying/Receiving other assistance programs (e.g., requesting some free samples from providers)

Definition: Patients may explicitly or implicitly mention that they are receiving (received), or they applied or plan to apply for financial assistance for a drug/device/supply from other sources, such as health care providers or a charity organization at a medical center.

Examples:

- “Especially since you had the Problem with the insulin and the hospital helped at that time.”
- “Most hospitals Have Patient advocates who should be able to tell you where to turn for help.”
- “My doctor wrote me an Rx for the strips and a letter to my insurance company for me to test up to 6 times a day, first thing a.m., before lunch, before supper, and before bedtime are the prime times for me.”

6. Discussing Non-adherence Behavior to Reduce Cost

6.1 Reducing the usage of the prescription drugs/devices/supplies

Definition: Patients may explicitly or implicitly state that they reduced the consumption of drugs/devices/supplies because of financial difficulties to access the drugs/devices/supplies.

Examples:

- “We used to cut the strips down the middle to save money.”
- “I have to be very conservative with my strips also.”

6.2 Skipping prescription drugs/devices/supplies

Definition: Patients may explicitly or implicitly state that they skipped drugs/devices/supplies because of financial difficulties to access drugs/devices/supplies.

Examples:

- “But I had a week where I didn't have any insulin at all a couple weeks back.”

6.3 Delay refilling the prescription drugs/devices/supplies filled

Definition: Patients may explicitly or implicitly state that they delayed refilling the prescribed drugs/devices/supplies because of financial difficulties to access drugs/devices/supplies.

Examples:

- “Will try to get one next weekend and some starter strips at the Diabetes Expo.”
- “So No test strips till my Drs visit Tuesdays.”

6.4 Stopped usage of the prescription drugs/devices/supplies

Definition: Patients may explicitly or implicitly state that they stopped refilling the prescribed drugs/devices/supplies because of financial difficulties to access the prescribed drugs/devices/supplies.

Examples:

- "He was walking around with 500+ blood sugar for two years, we could not afford to get it." (*In this sentence, the patient implicitly state that they stopped using insulin to control blood sugar*).
- "I have been diabetic for a very long time and at one point had no way to pay for medical care of any sort... I went without treatment, without my insulin, for three months, and I'm paying for it now with all kinds of complications."

7. Exchanging/Seeking Information

7.1 Seeking information for drugs/devices/supplies/services

Definition: Patients may explicitly or implicitly request information from other participants in the forum to access low price or free drugs/devices/supplies/services. Patients may also look for other type of health-related information, such as quality of treatment, specific diet to control symptoms.

Examples:

- "Wonder how many waves of the arm you can have in a ten supply?"
- "Insurance won't cover a cgm. Looking for enlite sensors or dexcom sensors and a dexcom share receiver."
- "Searching for a box of the quick set 9mm 23 inches or similar."
- "So my next question is - do you think if I put a new battery in my OneTouch Ultra meter that is 12 years old and get new test strips that I will get reliable readings?"

7.2 Exchanging information for drugs/devices/supplies/services

Definition: Patients may share their experience with accessing to low price or free drugs/devices/supplies/services with other participants in the forum. Patients may also share other health-related experiences with participants in the forums or recommend them to follow special activities to promote their health status.

Examples:

- "I know that most test strip and insulin makers offer copay cards, where u pay no more than like 25.00 monthly for supplies."
- "I recommend shopping for the cheapest test strips in bulk and buy the netter accordingly."
- "The company makes their money off the strips, which I won't lie, they are expensive (but insurance covers them)."
- "Did you know that you can usually get meters for free?"

8. Seeking/Offering Emotional Support

Definition: Patients may explicitly or implicitly provides emotional support or feel sympathy for other patients having problem with health status or access to drugs/devices/supplies/services.

Examples:

- “Hi Lady Mary, I was so sorry to hear about Medicare not paying for you to use more than one test strip a day.”
- “I really feel sorry for you.”
- “I also wanted to say it's great that you are supporting and helping your Mom through diabetes treatment.”
