# Supplementary material for: A Systematic Framework for Analyzing Patient-Generated Narrative Data: Protocol for a Content Analysis
Source: JMIR Res Protoc. 2019 Aug 26;8(8):e13914. doi: 10.2196/13914 (PMC6786846; doi:10.2196/13914)
Supplement: Multimedia Appendix 10 [file resprot_v8i8e13914_app10.pdf]

Multimedia Appendix 10: Final Analytical Framework

Final Themes with operational definition and example from patients comments for the case study "Identifying the underlying factors affecting patient attitudes toward antidepressants"

Source: Zolnoori, M. (2017). "Utilizing Consumer Health Posts for Pharmacovigilance: Identifying Underlying Factors Associated with Patients' Attitudes Towards Antidepressants." Theses and Dissertations. 1733. https://dc.uwm.edu/etd/1733

Code	Sub-codes	Description	Example
Adverse Drug Reactions	Presence	If the patient reported explicitly, he experienced adverse drug reactions (ADRs) with or without listing the ADRs in the sentence/comments.	"I couldn't take Effexor XR. It gave me horrible nightmares and I kept waking up."
	Absence	If the patients reported, they did not experience any ADRs.	"I did not have any side-effect."
Perceived distress from ADRs (ADR-PD)	High	- **Explicit mentions:** If the patient explicitly mentioned that they suffered from ADRs.<br>- **Functional problems:** If the patient reported functional problems associated with ADR, such as limitation in daily functioning, social activities, and work performance<br>- **Qualifiers indicating severity:** If the patient used any qualifiers indicating the severity of the symptoms, such as "severe," "debilitating," "intolerable."<br>- **Severe ADRs:** If the patient reported severe ADRs having the negative impact on the patient's quality of life including suicidal ideation/attempt, self-harm, bed-ridden. Report of hospitalization or emergency visit also shows the presences of ADRs causing high-perceived distress.	"The side effects are intolerable."<br>"Have been able to work (software developer) if attempting this drug during work week."<br>"Severe nausea and dizziness."<br>"That drug caused nausea and increased suicidal thoughts."
	Low	**Explicit mentions:** If the patient explicitly mentioned that the ADRs were tolerable.<br>**Qualifiers indicating mildness:** using Qualifiers indicating mildness of ADRs, such as slightly, mild.<br>**Qualifiers are indicating non-persistency:** Using qualifiers indicating non-persistency of ADS, given that ADRs are NOT associated with qualifiers indicating the severity of ADRs.<br>**No experience of ADRs:** If patient explicitly mentioned they did not experience any ADRs.	"Any side effects were, for me, tolerable compared to the benefits."<br>"Mild headache"<br>"Headache for two days, but severe headache for two days indicates high-perceived distress."<br>"The withdrawal made me very dizzy."
Withdrawal symptoms (WDs)	Presence	If the patient complained about occurring new symptoms in the process of dosage reduction, discontinuation, or missing dosages (unintentional withdrawal) of the medication, with or without listing the WDs symptoms.	"I weaned slowly from 150mg to 75 to 37.5 and off. I feel nauseous alot and my depression and social anxiety has returned almost 100%."<br>"Do not wean off effexor too soon as i had one very bad day."
	Absence	If the patient reported, they did not experience any withdrawal symptom.	

WD-perceived distress (WD-PD)	High	If the patient mentioned a) they suffered from withdrawal symptoms, and b) they reported functional problems associated with the WDs, and/or c) they used qualifiers indicating the severity of a specific WD, and/or d) they mentioned severe WDs, the WD is high.	"The withdrawal symptoms are horrible."<br>"I was in bed for about one week."<br>"I missed a dose yesterday, and now I'm nauseous."<br>" I can not function. Feel I am poisoned."
	Low	a) If the patient explicitly mentioned that withdrawal symptoms were tolerable, b) used indicators showing low perceived distress, c) used qualifiers showing tolerability of the symptoms, d) using qualifiers showing non-persistency of the symptoms, given that the symptom was tolerable, e) explicit mention of no experience of withdrawal symptoms.	"Withdrawal was fine."<br>"When I stopped the drug, I had mild dizziness."<br>"I experienced headache for few days after reducing the dosage."<br>"I had no experience of withdrawal symptoms."
Effectiveness (EF)		A drug is effective, if the patient reported that his health condition has been improved or his symptoms were treated after drug consumption.	"For the first few weeks it helped me feel better."
Ineffectiveness (INF)		A drug is ineffective if the patient reported that his/her health status did not improve, became worse, or still has the same symptoms.	"It did not help me at all."
Patient – physician interaction (PPI)	Positive (P)	A patient physician interaction is positive, if the patient expressed explicitly or implicitly their satisfactions from communications with clinicians.	"Success with these meds truly depends on staying in touch with your physician" *(Implying trust in the clinician).*<br><br>"My doctor and I decided to stop taking it" *(implying the clinician involved the patient in the process of decision making that can lead to patient satisfaction).*
	Negative (N)	- If the patient expresses explicitly or implicitly their dissatisfactions from communications with clinicians, a patient physician interaction is negative.<br>- The patient may complain about provider's failure in providing sufficient information or non-effective communication, such as provider's failure to involve the patient in the process of decision-making or the treatment plan.	"Dr. s do not understand the crazy side effects of starting this class of drugs" *(Implying patient's complaint about physician's lack of knowledge).*<br>"The doctor still claims that 30 mg is not even considered a therapeutic dose, but I know what works" *(physician failure to include patient treatment preference in the process of decision making).*
Lack of knowledge (~KN)		- Some patients may complain that they did not receive sufficient information about ADRs or WDs symptom associated with the drug and the mechanism of management of the ADRs/WDs.<br>- The patient may indirectly complain about lack of knowledge by asking questions in the forum or mentioning that they did search on the web to gain more information about the drug.	"Cannot get straight answer from anyone regarding how long these withdrawal symptoms will last."<br>"No one informed me of the withdrawal nightmare."<br>"TODAY I'd like to know if my recent muscular twitches are related to this medicine."<br>"I wish I had been smart enough to do research on Effexor BEFORE I went on it."
	Unintentional WD (DXD-F)	If the patient explicitly mentioned that they forgot to take medication (missing dosages) or run out of medication, the discontinuation is unintentional.	"When I miss a day I feel very spaced out, thick, groggy, sad."<br>"If I don't take the medicine at the same time every day or forget a day, I will

			experience withdraw and its horrible!”
Experience of WD	Intentional WD-Stopping (DXD-S)	If the patient explicitly mentioned that they stopped (discontinue) the medication, the discontinuation is intentional and should be labeled as DXD-S.	“I had to stop taking it.”<br>“I had low blood pressure for about 2 weeks after I stopped taking it.”
	Intentional WD-weaning off (DXD-W)	If the patient explicitly mentioned that they are weaning off (tapering off) the medication. The discontinuation is intentional and should be labeled as DXD-W.	“I have been tapering from 60 mg per day.”<br>“I'm stopping slowly.”
	Intentional decision for WD (DXD-Dec)	If the patient explicitly mentioned that they decided to stop or wean off the medication, the sentence should be labeled as DXD-Dec.	“However after dealing with this acne I'm going to try another med.”
Dosage/duration		If the patient talked about dosage or duration of the drug, it will be labeled as positive.	“My doctor prescribed 25 ml for me.”
