# Supplementary material for: A Systematic Framework for Analyzing Patient-Generated Narrative Data: Protocol for a Content Analysis
Source: JMIR Res Protoc. 2019 Aug 26;8(8):e13914. doi: 10.2196/13914 (PMC6786846; doi:10.2196/13914)
Supplement: Multimedia Appendix 11 [file resprot_v8i8e13914_app11.pdf]

Multimedia Appendix 11- Interpretation of the obtained results from the Cohen Kappa

Definition of kappa is as follows:

$$k = \frac{P(a) - P(e)}{1 - P(e)}$$

where P(a) denotes the observed percentage of agreement, and P(e) denotes the probability of expected agreement due to chance [1, 2]. Possible values for the Kappa coefficient ranges from -1 to 1, where 1 indicates complete agreement, 0 indicates completely random agreement, and -1 indicates complete disagreement. Landis and Koch [3] provides more specific instruction on the obtained results from kappa: 0.0 to 0.2 indicates "slight agreement," 0.21 to 0.40 indicates "fair agreement," 0.41 to 0.60 indicates "moderate agreement," 0.61 to 0.80 indicates "substantial agreement," and 0.81 to 1.0 indicates "almost perfect" or "perfect agreement". Low IAA means that the annotators found it difficult to agree on which unit of analysis belonged to a theme and which did not. The theme may be interesting from the perspective of qualitative analysis, but cautions needs to be taken when including the theme in the analysis of finding. According to Hayes and Krippendorff, themes with k less than 0.67 should be discounted from the analysis, themes (variables) with k between 0.67 and 0.80 should be tentatively used in the analysis, and themes with k above 0.80 can be used for the definite conclusion[4]. In contrary to this instruction, in practice, themes with k less than 0.67 is often retained in the conclusion of the studies. Taking into account this instruction strongly depends on a study's research question and methodology.

We used Cohen Kappa to compute the inter annotator agreement (IAA) for the project "attitudes to antidepressants." The obtained Kappa for this case study was 0.78, indicating a substantial agreement between annotators.

1. Hallgren KA. Computing Inter-Rater Reliability for Observational Data: An Overview and Tutorial. Tutorials in quantitative methods for psychology. 2012;8(1):23-34. PMID: PMC3402032.
2. Artstein R, Poesio M. Inter-coder agreement for computational linguistics. Computational Linguistics. 2008;34(4):555-96.
3. Landis JR, Koch GG. The measurement of observer agreement for categorical data. biometrics. 1977:159-74.
4. Hayes AF, Krippendorff K. Answering the call for a standard reliability measure for coding data. Communication methods and measures. 2007;1(1):77-89.
