# Supplementary material for: A Systematic Framework for Analyzing Patient-Generated Narrative Data: Protocol for a Content Analysis
Source: JMIR Res Protoc. 2019 Aug 26;8(8):e13914. doi: 10.2196/13914 (PMC6786846; doi:10.2196/13914)
Supplement: Multimedia Appendix 12 [file resprot_v8i8e13914_app12.pdf]

Multimedia Appendix 12: Descriptive interpretation of the findings of the case study "access to diabetes medications"

"We identified 7 major themes and 20 sub-themes in the sampled patient posts. The most frequent theme was seeking or exchanging information, followed by non-adherence behavior, which includes skipping, delaying, or reducing the prescribed dosages. Around 20% of the patients shared information about financial assistance programs from public organizations or pharmaceutical companies for low-cost insulin purchase. Some other patients reported using herbal supplement or cheaper alternative drugs other than insulin to lower blood glucose level. Additional patients changed their lifestyle, by using low-carbohydrate diets or exercising more to manage their blood glucose. To our surprise, some patients also bought insulin from non-conventional channels such as eBay or overseas, and traded or borrowed insulin from peer patients."
