# Supplementary material for: A Systematic Framework for Analyzing Patient-Generated Narrative Data: Protocol for a Content Analysis
Source: JMIR Res Protoc. 2019 Aug 26;8(8):e13914. doi: 10.2196/13914 (PMC6786846; doi:10.2196/13914)
Supplement: Multimedia Appendix 13 [file resprot_v8i8e13914_app13.pdf]

Multimedia Appendix 13: The major difference between the systematic framework and other content analysis framework suggested by other studies.

This systematic framework is different from other frameworks suggested for content analysis such as "Theme development in qualitative content analysis and thematic analysis"[1], "The qualitative content analysis process" [2], and "How to plan and perform a qualitative study using content analysis"[3] from the following aspects:

1) This systematic framework was specifically designed to convert patients' reported experiences in online healthcare forums (communities) to meaningful concepts, highlighting various dimensions of the dataset related to the research questions. However, the focuses of other content analysis frameworks are on other types of qualitative data collected using other methods, such as observation of situations, interview, and focus groups.

2) In this systematic framework, we suggested text-mining methods to facilitate and accelerate the process of data collection and preparation. Other content analysis framework did not incorporate text-mining methods in the process of content analysis.

3) In this systematic framework, we showed that how combining the Framework Method and the deductive-inductive approach can speed up the process of data analysis and facilitate interpretation of the results. Other content analysis framework did not follow the same approach for the content analysis of patients' narrative.

4) We demonstrated the procedure of implementation of this systematic framework using two research questions with a focus on patients' treatment experiences reported in online healthcare forums. We showed the procedure of data collection, data preparation, data analysis and analysis of findings for the two questions. Other content analysis framework did not use online healthcare forums to demonstrate the procedure of content analysis.

1. Vaismoradi M, Jones J, Turunen H, Snelgrove S. Theme development in qualitative content analysis and thematic analysis. 2016.
2. Elo S, Kyngäs H. The qualitative content analysis process. Journal of advanced nursing. 2008;62(1):107-15.
3. Bengtsson M. How to plan and perform a qualitative study using content analysis. NursingPlus Open. 2016;2:8-14.
